# Rapid Scan Electron Paramagnetic Resonance Spectroscopy Is a Suitable Tool to Study Intermolecular Interactions of Intrinsically Disordered Protein

**DOI:** 10.3390/biology12010079

**Published:** 2023-01-03

**Authors:** Jessica Dröden, Malte Drescher

**Affiliations:** Department of Chemistry, Konstanz Research School Chemical Biology, University of Konstanz, 78457 Konstanz, Germany

**Keywords:** aggregation, α-synuclein (aS), electron paramagnetic resonance (EPR) spectroscopy, intermolecular interactions, intrinsically disordered protein (IDP), rapid scan (RS)

## Abstract

**Simple Summary:**

To understand complex cellular processes, the investigation of intrinsically disordered proteins that are involved in the most crucial mechanisms is of utmost importance. However, intrinsically disordered proteins lack a well-defined structure and exhibit vast flexibility. A prominent example is α-synuclein, that under pathological conditions, self-interacts and forms highly-ordered filamentous aggregates characteristic of neurodegenerative disorders, e.g., Parkinson’s disease. Due to its broad conformational ensemble, its investigation is hampered, and suitable biophysical methods to elucidate underlying interactions and mechanisms on a molecular level are scarce. Investigating the aggregation process and prevailing kinetics is essential to gain insight into the causes of the pathological processes and eventually invent effective treatments. Here, we set out to investigate the accelerated aggregation process of α-synuclein and its disease mutants in the presence of ethanol. For the first time, we made use of rapid scan electron paramagnetic resonance spectroscopy to study the kinetics of α-synuclein aggregation. We were able to highlight differences between different protein variants and demonstrated that this approach outperforms conventional techniques in terms of sensitivity and rapidity of data acquisition and successfully demonstrated that this technique is suitable for studying intermolecular interactions with fast kinetics.

**Abstract:**

Intrinsically disordered proteins (IDPs) are involved in most crucial cellular processes. However, they lack a well-defined fold hampering the investigation of their structural ensemble and interactions. Suitable biophysical methods able to manage their inherent flexibility and broad conformational ensemble are scarce. Here, we used rapid scan (RS) electron paramagnetic resonance (EPR) spectroscopy to study the intermolecular interactions of the IDP α-synuclein (aS). aS aggregation and fibril deposition is the hallmark of Parkinson’s disease, and specific point mutations, among them A30P and A53T, were linked to the early onset of the disease. To understand the pathological processes, research intensively investigates aS aggregation kinetics, which was reported to be accelerated in the presence of ethanol. Conventional techniques fail to capture these fast processes due to their limited time resolution and, thus, lose kinetic information. We have demonstrated that RS EPR spectroscopy is suitable for studying aS aggregation by resolving underlying kinetics and highlighting differences in fibrillization behavior. RS EPR spectroscopy outperforms traditional EPR methods in terms of sensitivity by a factor of 5 in our case while significantly reducing data acquisition time. Thus, we were able to sample short time intervals capturing single events taking place during the aggregation process. Further studies will therefore be able to shed light on biological processes proceeding on fast time scales.

## 1. Introduction

To fully understand complex cellular processes, proteins should not be considered isolated species; rather, they constitute key players in sophisticated interaction networks [1]. Intrinsically disordered proteins (IDPs) usually form hubs in these networks interacting with a multitude of partners [2,3]. IDPs lack a well-defined three-dimensional structure; however, they are shown to undergo disorder-to-order transitions upon interaction [4]. Their inherent flexibility and heterogeneous conformational ensemble complicate their investigation, rendering them unattainable for high-resolution techniques.

A prominent example is the IDP α-synuclein (aS) that, upon interaction with negatively charged membranes, exhibits an α-helical structure [5,6]. Under pathological conditions, aS self-interacts forming non-functional, highly-ordered filamentous aggregates that accumulate in Lewy bodies that are characteristic of Parkinson’s disease (PD) and related neurodegenerative disorders [7,8]. Research intensively focuses on understanding the process and kinetics of aS aggregation and searching for parameters that influence the aggregation. Reported models postulate the formation of oligomers constituting the toxic species with fibrils representing the end-point of the process [9,10,11,12].

Early onset PD was linked to several point mutations in the gene encoding aS. Among others, the mutations A30P [13] and A53T [14] are particularly interesting, and their aggregation behavior was investigated thoroughly. These studies discovered faster aggregation for aS A53T compared to the aS wild type (wt) [15,16], whereas for aS A30P varying findings ranging from slower to faster kinetics were reported [16,17,18,19].

Besides alteration in the primary sequence of aS, the environment strongly influences the kinetics of aggregation. Not only the pH and the salt concentration of the buffer [20,21] showed an impact, but also the presence of lipids [21,22] or organic solvents such as ethanol (EtOH) [23,24,25] altered the kinetics of fibrillization.

Conventional techniques to investigate protein aggregation include Thioflavin T fluorescence, transmission electron microscopy (TEM), atomic force microscopy, and circular dichroism (CD) spectroscopy. In addition, site-directed spin labeling (SDSL), in combination with electron paramagnetic resonance (EPR) spectroscopy [26,27], was shown to be a powerful tool for studying aS aggregation and the structure of resulting fibrils [28,29,30,31,32]. Conventional continuous wave (CW) EPR experiments probe local side chain dynamics of nitroxide spin labels reflected in the spectral shape and report on aggregation progression. Using this approach, Zurlo et al. were able to reveal the aggregation kinetics of singly spin-labeled aS [33,34]. However, spectra acquisition time was considerably long (3 to 8 h depending on the sample), necessitating the intermediate storage of sample aliquots and waiting time. Thus, this approach rules out the possibility of investigating unstable, non-storable samples (e.g., short-lived intermediates) or detecting fast kinetics.

To overcome these limitations, we used rapid scan (RS) EPR spectroscopy [35,36]. We have already successfully used this technology to study aS-lipid interaction, where changes in the spectral shape accounted for the binding of aS to lipid membranes revealing their interaction even inside living cells [37]. Here, we set out to investigate even the accelerated aggregation of aS in the presence of EtOH and the differences between the aggregation of aS variants bearing different point mutations. Using RS EPR spectroscopy, we significantly reduced spectra acquisition time, while the obtained signal-to-noise ratio (SNR) still outperforms the conventional CW EPR approach without affecting spectral resolution [38]. Ultimately, RS EPR spectroscopy allows capturing the kinetics of fast biological processes, which were so far not accessible on a second-time scale by EPR.

## 2. Materials and Methods

### 2.1. Protein Production and Spin Labeling

Plasmid mutagenesis, protein expression, and purification were performed as described previously [37,39]. Here, a single cysteine residue was introduced in aS variants at position 27, enabling spin labeling with thiol-reactive nitroxide. Prior to labeling, aS cysteine mutant samples were incubated with 2 mM 1,4-dithiothreitol (DTT) for 30 min at room temperature to resolve disulfide bonds that possibly formed during storage and thawing. DTT was removed using two desalting steps (Zeba™ Spin Desalting Resin, 7 MWKO, Thermo Fisher Scientific, Waltham, MA, USA). Subsequently, a 6-fold molar excess of the methanethiosulfonate spin label MTSSL (Enzo Life Sciences, Lörrach, Germany) was added to the protein sample and incubated overnight at 4 °C in an overhead rotor. The excess spin label was removed by one desalting step using a HiPrep 26/10 desalting column (GE Healthcare, Uppsala, Sweden) installed on the ÄKTAprime plus chromatography system. Within this step, the buffer was changed to aS aggregation buffer (10 mM Tris, pH 7.4, 150 mM NaCl). The success of labeling was assessed by measuring CW EPR spectra. The final protein concentration was determined spectrophotometrically by UV absorption at 280 nm. The labeling efficiency was determined to be >90% for all mutants. Samples were stored at −80 °C.

### 2.2. Aggregation Assay

aS aggregation was performed as described previously [23]. Monomeric aS with a final concentration of 35 µM in aS aggregation buffer + 20% (*v*/*v*) EtOH was stirred with a micro-stirring bar in a glass vial at 37 °C and 600 rpm. At regular intervals, aliquots of 20 µL (for EPR and TEM experiments) or 60 µL (for CD experiments) were taken and measured immediately (except TEM). RS EPR and CD experiments cover time points 0, 5, 10, 20, 40, 60, 120, 180, 360, and 1440 min, whereas CW EPR spectra captured the time points 0, 20, 60, 120, 180, 360, and 1440 min due to need for longer spectra acquisition time.

### 2.3. Rapid Scan EPR Spectroscopy

Samples with a volume of 20 µL were filled in glass capillaries (HIRSCHMANN^®^ ringcaps^®^, inner diameter 1.02 mm, Eberstadt, Germany) and sealed with Hemato-Seal™ capillary sealant (Fischer-brand™, Schwerte, Germany). RS spectra were recorded on an Elexsys 500 spectrometer (Bruker, Karlsruhe, Germany) equipped with the Rapid-Scan Accessory (Bruker, Karlsruhe, Germany) [40] at X-band frequency (9.426 GHz). Sinusoidal rapid magnetic-field scans at a frequency of 20 kHz with a scan width of 20 mT were applied using a 1D field experiment. The center field was set to 336.4 mT, the attenuation to 20 dB (2 mW power), and the background correction function TwoTone included in the XEpr software (Bruker) was chosen. The measurement time was set to 60 s resulting in averaging of 1,202,643 scans (63,297 onboard averages, 19 off-board averages). Recorded spectra were processed with Matlab R2019b (The Mathworks, Inc., Natick, MA, USA) and the toolbox EasySpin 6.00 [41]. Processing included baseline correction, adjusting the field position of the spectra to correct for small deviations in the microwave frequency between different samples, smoothing of data with a Savitzky–Golay filter function, and the normalization of the intensity.

## 3. Results

### 3.1. Spin-Labeled α-Synuclein Variants Aggregate in the Presence of Ethanol

**Figure 1:**
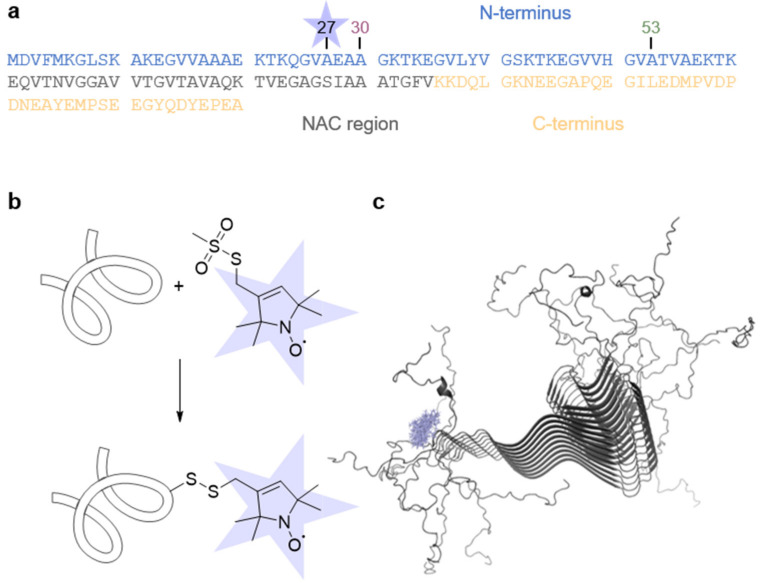
Spin labeling of aS. (**a**) aS primary sequence. The different regions of aS are highlighted. The position of the spin label (blue star) and the disease point mutations A30P and A53T are indicated. (**b**) Labeling scheme of aS with MTSSL spin-label. (**c**) Solid-state NMR fibril structure (PDB ID 2N0A [45]) and MTSSL rotamers (blue) at position 27.

aS consists of 140 amino acid residues and can be divided into different regions: a positively charged N-terminus, including an amyloidogenic hydrophobic NAC region, and a highly negatively charged C-terminus (Figure 1a) [42,43,44]. To demonstrate the versatility of our method, besides the wt protein, we used the aS disease variants A30P and A53T, for which the point mutations were reported to impact pathological processes [15,17,18]. Since aS is intrinsically diamagnetic, we introduced a single cysteine residue at position 27 to enable attachment of the thiol-specific methanethiosulfonate spin label (MTSSL) via SDSL (Figure 1b), reporting on the local side chain dynamics.

All aS variants with a concentration of 35 µM were independently allowed to aggregate at pH 7.4 in the presence of 20% EtOH at 37 °C and 600 rpm stirring for 1440 min (corresponds to 24 h). On a molecular level, aS fibrils consist of a highly ordered, β-sheet rich core (residues 36 to 98) exhibiting a parallel in-register arrangement, whereas the N- and C-termini remain flexible and unstructured [28,29,31,45,46,47,48]. Figure 1c shows the high-resolution structure of aS fibrils with calculated MTSSL rotamers attached at position 27.

First, we verified that the cysteine mutagenesis and attachment of MTSSL do not influence the morphology (Figure S1) nor the fibrils’ secondary structure (Figure S2). We found that spin-labeled aS forms a β-sheet structure and highly ordered fibrils in the presence of EtOH, concluding that under our conditions, the aggregation is undisturbed.

### 3.2. Spin-Dilution Is Not a Prerequisite in Our Experimental Setup

Spin labels placed in close proximity, e.g., in fibrils, interact with each other, impacting the EPR spectrum’s line shape. Thus, diamagnetic dilution is a frequently used tool so that spectral changes solely result from differences in spin mobility. Figure 1c clearly shows that the spin label at position 27 is expected to be located outside the β-sheet core of the fibril, and no effect of the spin–spin interaction between labels within the fibril is expected. For experimental proof, we compared the EPR spectra of aS monomer and fibril recorded at 120 K (Figure S3). We found no spectral broadening in the fibril spectrum compared to monomeric aS species and concluded that no diamagnetic dilution is required in our experimental setup. Hence, we improved our SNR due to the increased spin concentration in our sample.

### 3.3. Circular Dichroism Reveals the Global Aggregation Process of α-Synuclein

Next, we monitored the aggregation process of the spin-labeled aS variants within the time range of 1440 min by CD spectroscopy (Figure S4). This technique reports on the overall secondary structure of the protein backbone, thus reporting on global structural changes. Here, the spectra reveal a transition from a random coil secondary structure to a β-sheet structure with time. After 1440 min, each aS variant exhibits solely β-sheet content representing the end-point of aggregation. A qualitative comparison of the spectra of the different variants indicates that the kinetics of the global structural transition differ between the aS variants, with aS A30P-A27C-MTSSL tending to convert slowest.

### 3.4. The Signal-to-Noise Ratio of Rapid Scan EPR Spectroscopy Outperforms Conventional Continuous Wave EPR Spectroscopy

To assess the process of aS aggregation locally, with respect to the spin-labeled site, we measured time-resolved CW spectra at room temperature (Figure S5). Clearly, the spectral broadening is visible over the course of 1440 min. In order to obtain a decent SNR, we accumulated 30 spectra, each recorded for 1 min resulting in a total measurement time of 30 min for each spectrum. Thus, we can sample only one single time point every 30 min. Obviously, we are able to probe smaller intervals in real-time compared to the reported studies [33,34]. However, the CD spectra clearly show global changes taking place even within the first half hour, underlining that we lack sufficient time resolution when performing conventional CW EPR experiments.

**Figure 2:**
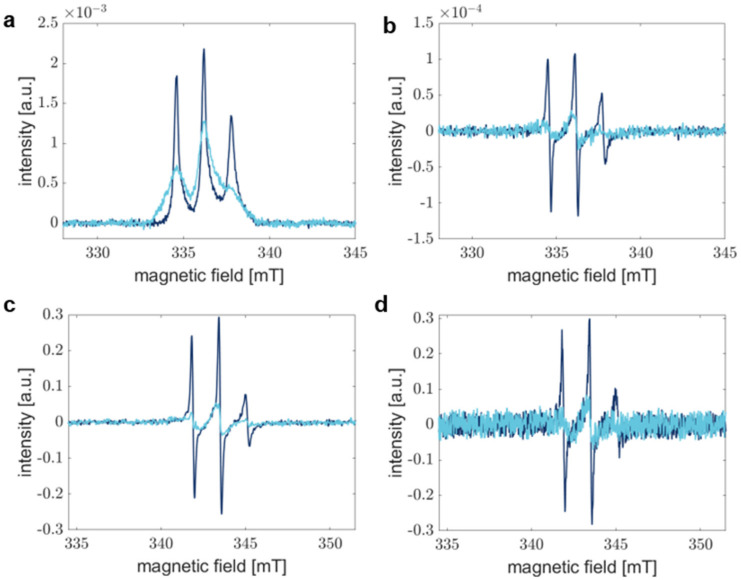
Comparison of the sensitivity of RS and CW EPR experiments. Dark blue spectra correspond to aS A27C-MTSSL monomer sample, whereas light blue spectra show aS in fibrils. (**a**) Absorption spectra recorded by RS EPR spectroscopy were accumulated for 1 min. (**b**) Pseudo-modulated RS EPR spectra from (**a**) modulated with 0.1 mT. (**c**) CW EPR spectra after 30 min of acquisition and averaging. (**d**) CW EPR spectra represent accumulation for 1 min. Field modulation in CW (**c**,**d**) results in the detection of the first derivative of the absorption spectrum.

Here, RS EPR spectroscopy offers advantages by shortening the measurement time without sacrificing SNR [49]. Figure 2 compares RS EPR spectra acquired for 1 min (Figure 2a) and pseudo-modulated RS EPR spectra (Figure 2b) with conventional CW EPR spectra accumulated for 30 min (Figure 2c) and 1 min (Figure 2d), respectively. The SNR in CW EPR spectra drops drastically, especially if spectra are broad, as is the case for spectra acquired after 1440 min of aggregation. In this regard, slight spectral differences are not distinguishable anymore, and CW EPR fails to report on precise processes. Under our experimental conditions, a typical RS EPR experiment exhibits an SNR improved by a factor of approximately five compared to CW EPR when samples are measured for 1 min in both experiments (see Table 1, monomer).

### 3.5. Rapid Scan Experiments Capture the Local Kinetics of α-Synuclein Aggregation

Exploiting reduced acquisition time while improving the SNR, we monitored the aggregation of the aS variants by RS EPR spectroscopy. Here, we were able to sample more time points in shorter time intervals compared to CW EPR experiments (see Section 2.2). Especially at the beginning of the aggregation process, where CD spectra suggested slight global secondary structural changes, we probed short intervals to examine transitions on a locally resolved level.

**Figure 3:**
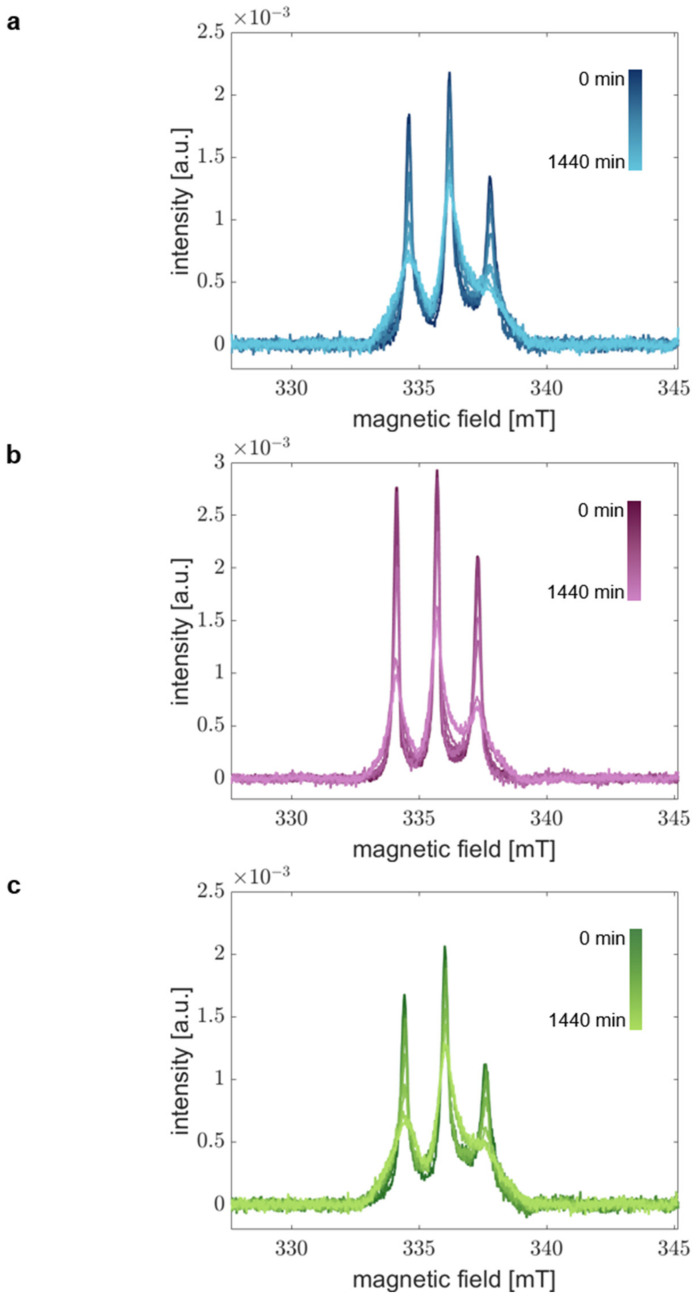
Time-resolved absorption spectra demonstrate aggregation of aS. Room-temperature RS EPR spectra of (**a**) aS A27C-MTSSL, (**b**) aS A30P-A27C-MTSSL, and (**c**) aS A53T-A27C-MTSSL. aS was aggregated at 37 °C and 600 rpm in the presence of 20% EtOH. Samples at different time points in the range of 0–1440 min were acquired in real-time.

Figure 3 shows the obtained time-dependent RS EPR spectra for all aS variants in the time range of 0 to 1440 min. We clearly find changes in the spectral shape over time. As expected, the spin mobility decreases due to the aggregation’s progression resulting in the spectra’s broadening. We find a systematic trend of spectral broadening for all variants and obtain similar spectra after 1440 min in all cases. Thus, we conclude that we could describe the complete course of aS aggregation from monomer to fibril.

Considering the spectral changes with time, we find different aggregation kinetics for the individual aS variants. This finding agrees with the CD spectra indicating differences in the kinetics of global structural transitions. These results infer that the local structural changes in the extremal N-terminal region occur on the same time scales as the overall aS backbone transitions and different kinetics of each aS variant are displayed by our experimental approach.

Thus, we demonstrated that RS EPR spectroscopy could monitor the complete aggregation process of different aS variants in a time- and site-resolved manner, even though the kinetics are accelerated in the presence of 20% EtOH.

## 4. Discussion

Biophysical methods to study protein aggregation are important to understanding neurodegenerative disease pathological processes. Here, we used rapid scan EPR spectroscopy to investigate the aggregation of the IDP aS implicated in PD, whose fibrillization and deposition in Lewy bodies causes a decline in motor and cognitive functions [7,50]. While its implication in pathogenesis is indisputably approved, the physiological role of aS is still not fully understood. However, the involvement in membrane remodeling and vesicle trafficking [51,52,53] is thought to be associated with its function.

Further development of the rapid scan technique enables the detection of a broad magnetic field range (~200 G), allowing for the complete acquisition of spectra of nitroxide spin labels. Previously, we exploited this feature of RS EPR spectroscopy to investigate aS-lipid interaction [37] to gain insight into aS functioning inside cells. Here, we set out to investigate the aggregation of different aS variants using the RS EPR approach, which, to our knowledge, has not yet been realized.

Under our experimental conditions, aS aggregation was accelerated due to the presence of 20% EtOH, which was shown to influence aS aggregation kinetics [23,24]. Here, the strength of the RS technique, able to acquire spectra with high time resolution while exhibiting a good SNR, can be exploited. Evaluating changes in the line shape that reflects the mobility of the attached MTSSL spin label was used to follow the course of aS aggregation on a minute time scale. Compared to conventional CW experiments that capture time intervals of 30 min, we gain information and can monitor every single event taking place during the aggregation process.

Over time, we find that decreased spin label mobility is reflected in broader spectral line shapes. Under our conditions, all aS variants aggregate into fibrils within 24 h, exhibiting different kinetics that agree with CD spectroscopy results. Thus, we demonstrated the suitability of RS EPR spectroscopy to detect the process of aS aggregation with high time and site resolution. Now, it is possible to label different positions within the aS sequence and perform an aggregation site scan. This will allow determining regions involved in different phases of the aggregation process, gaining new insights into pathological processes of neurodegenerative diseases.

## 5. Conclusions

We successfully applied RS EPR spectroscopy to monitor the intermolecular interaction of the IDP aS and demonstrated its suitability. We obtained a high time resolution while spectra were acquired in good quality with sufficient SNR. Future studies could exploit these advantages to investigate other time-resolved biological processes. Even faster aggregation kinetics, as reported, e.g., for the IDP Aβ, will be accessible with RS EPR spectroscopy, and these results might shed new light on crucial cellular processes, such as the pathological aggregation of proteins in neurodegenerative diseases.

## Figures and Tables

**Table 1:** Comparison of the signal-to-noise ratios of RS and CW EPR experiments with spectra acquired for 1 min. The SNR of the RS EPR spectra were determined prior to pseudo-modulation.

Accumulation Time	RS	CW
monomer	121	26
fibril	54	4

## Data Availability

Raw data is provided by the authors upon request.

## Supplementary Materials

The following supporting information can be downloaded at: https://www.mdpi.com/article/10.3390/biology12010079/s1, Supplementary experimental details, Figure S1: Comparison of aS fibril morphology in the presence of cysteine mutation and MTSSL spin label; Figure S2: Comparison of the aS secondary structure in the presence of cysteine mutation and MTSSL spin label; Figure S3: Low-temperature CW EPR spectra of aS A27C-MTSSL; Figure S4: Time-resolved CD spectra of the spin-labeled aS variants; Figure S5: Time-resolved CW EPR spectra of the spin-labeled aS variants.
