# Supplementary material for: Rapid Scan Electron Paramagnetic Resonance Spectroscopy Is a Suitable Tool to Study Intermolecular Interactions of Intrinsically Disordered Protein
Source: Biology (Basel). 2023 Jan 3;12(1):79. doi: 10.3390/biology12010079 (PMC9856040; doi:10.3390/biology12010079)
Supplement: Supplementary file 1 [file biology-12-00079-s001.zip › biology-2111028-supplementary.pdf]

Supporting information

Supplementary experimental details

EPR spectroscopy

Samples with a volume of 20 µL were filled into glass capillaries (HIRSCHMANN® ringcaps®, inner diameter 1.02 mm) and sealed with Hemato-Seal™ capillary tube sealant (Fischer-brand™).

Room-temperature continuous wave EPR spectroscopy. CW EPR spectra at room temperature were recorded at X-band frequency (9.645 GHz) with the benchtop spectrometer EMXnano (Bruker), equipped with a cylindrical cavity (mode TM_{110}). The center field was set to 343 mT using a sweep width of 20 mT and a sweep time of 60 s. The modulation amplitude was set to 0.1 mT, the microwave attenuation to 17 dB (2 mW power), and the modulation frequency to 100 kHz. 30 scans were averaged. To assess the spin labeling efficiency, the spin concentration was determined quantitatively using the built-in EMXnano reference-free spin counting module (Xenon software, Bruker) to estimate the amount of MTSSL.

Low-temperature continuous wave EPR spectroscopy. Low-temperature CW spectra were recorded at 120 K at X-band frequency (9.472 GHz) with the benchtop spectrometer MiniScope MS5000 (Freiberg Instruments GmbH/Bruker). The center field was set to 337.3 mT using a sweep width of 30 mT and a sweep time of 60 s. The modulation amplitude was set to 0.35 mT, the microwave attenuation to 33 dB (0.05 mW power), and the modulation frequency to 100 kHz. 60 scans were averaged.

EPR data processing. Recorded spectra were processed with Matlab R2019b (The Mathworks, Inc.) and the toolbox EasySpin 6.00 [1]. Processing included baseline correction, adjusting the field position of the spectra to correct for small deviations in the microwave frequency between different samples, smoothing of data with a Savitzky-Golay filter function, and normalization of the intensity.

Sensitivity comparison of continuous wave and rapid scan spectra. The SNR for both measuring modes was calculated by dividing the signal amplitude by the standard deviation of the noise level. To calculate the standard deviation of the noise level, the measured intensity in the range of 349 – 353 mT for CW, and 341.8 – 345.1 mT for RS, respectively was calculated. To allow comparison of the SNR in both measuring modes, we pseudo-modulated the RS spectra with an amplitude of 0.1 mT (equivalent to modulation amplitude in CW EPR measurements) using the software toolbox EasySpin 6.00 [1]. Pseudo-modulation of the absorption spectra measured in RS mode yields the first derivative of the spectral line shape, which corresponds to the line shape measured in CW EPR mode and thus, allows direct comparability.

Circular dichroism spectroscopy

CD measurements were recorded on a JASCO J-715 spectropolarimeter with 0.5 mm demountable quartz cuvettes (Hellma Analytics). CD samples were diluted to a volume of 100 µL with aS aggregation buffer (final protein concentration: 20 µM). All spectra were acquired at 20 °C using a continuous scanning mode with 1.0 nm bandwidth and 50 nm/min scanning speed. 5 scans per sample ranging from 180-280 nm in wavelength were averaged. Spectra were baseline-corrected and the background spectrum of the appropriate control sample was subtracted. The CD signal (spectrometer unit in mdeg) was converted to the molar residue

ellipticity (MRE) using the formula MRE = (MRW*CD signal/1000)/(10*d*c) with M in g/mol, N_{aa} as the number of amino acid residues in the protein, MRW = M/N_{aa}, c in g/mL, and d = 0.05 cm.

Electron microscopy

The samples were diluted with aS aggregation buffer to a final concentration of 20 µM. An aliquot of 5 µL was withdrawn from the samples and applied on a glow-discharged carbon-coated Formvar grid (Plano GmbH). After incubation for 5 min, the grid was washed with 5 times with H_2O bidest., and subsequently two times (1 min and 5 min, respectively) negatively stained with 2 % (w/v) phosphotungstic acid solution (Na-PTA), pH 7.6. The Na-PTA solution was freshly prepared in H_2O bidest., pH adjusted and filtered through 0.22 µM sterile syringe filters prior to use. TEM images were recorded digitally on a TEM Zeiss Omega 912 instrument operated at 80 kV using magnification in the range of 25,000-100,000x.

Supplementary figures

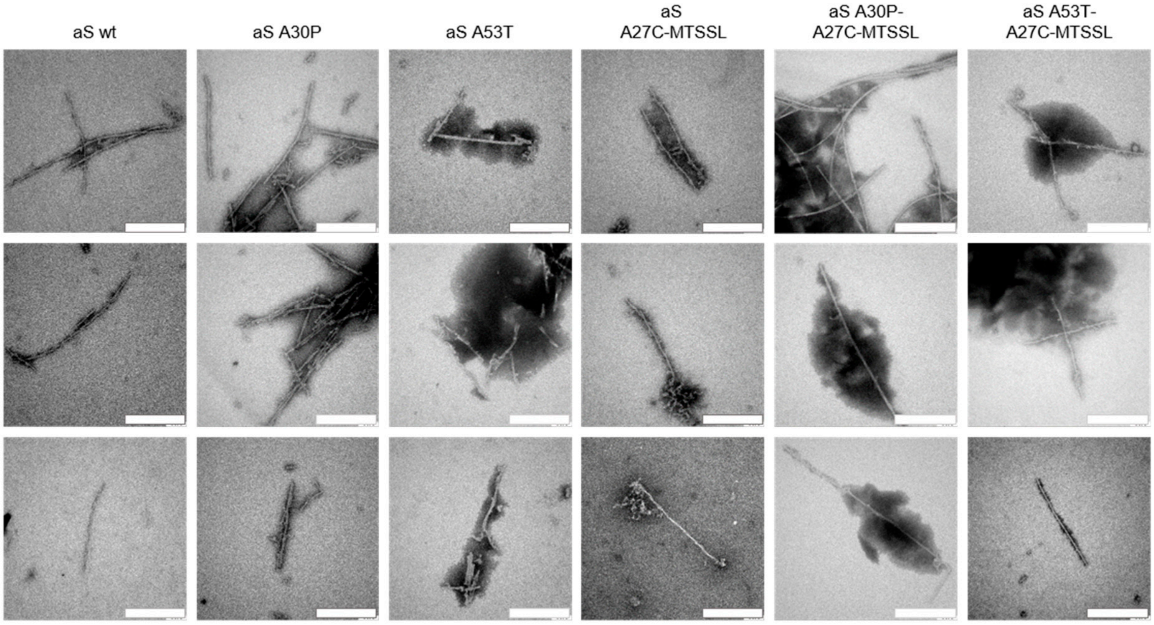

Figure S1. Comparison of aS fibril morphology in presence of cysteine mutation and MTSSL spin label. Each variant was allowed to aggregate at 37 °C and 600 rpm in presence of 20 % EtOH for 1440 min before visualization by TEM. All aS variants form fibrils of comparable structure demonstrating that mutagenesis and spin label attachment does not influence the product of aggregation. Scale bar: 300 nm.

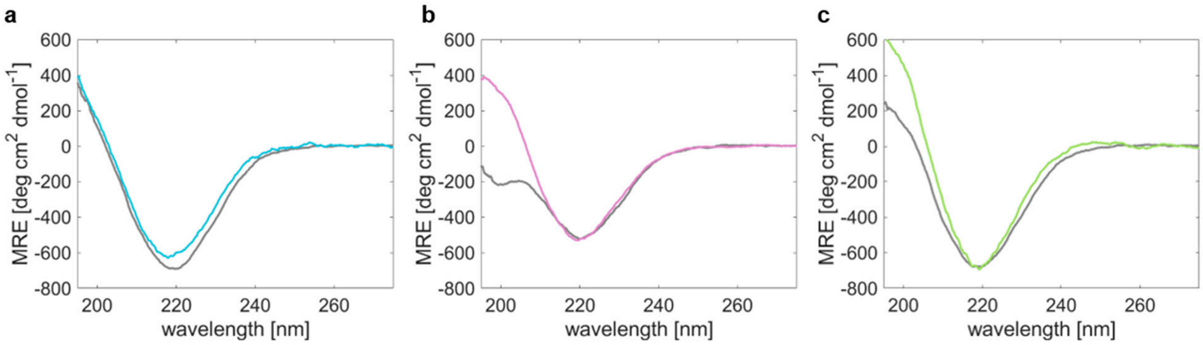

Figure S2. Comparison of aS secondary structure in presence of cysteine mutation and MTSSL spin label. Each variant was allowed to aggregate at 37 °C and 600 rpm in presence of 20 % EtOH for 1440 min before visualization by CD spectroscopy. (**a**) aS A27C-MTSSL (blue), (**b**) aS A30P-A27C-MTSSL (magenta), and (**c**) aS A53T-A27C-MTSSL (green). For comparison, the respective aS variants without the cysteine mutation and attached MTSSL spin label are shown in grey. In case of aS A30P, the conversion to β-sheet only is not complete after 1440 min. However, all aS variants form β-sheet secondary structure demonstrating that mutagenesis and spin label attachment does not significantly influence the product of aggregation.

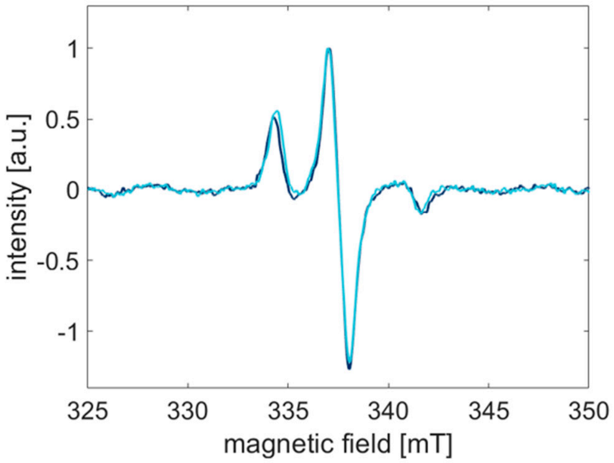

Figure S3. Low-temperature CW EPR spectra of aS A27C-MTSSL. The spectra of monomeric aS (dark blue) and after aggregation for 1440 min (light blue) were recorded at 120 K. No spectral broadening is visible showing that no diamagnetic dilution is required when spin label is placed outside of the fibril core (position 27).

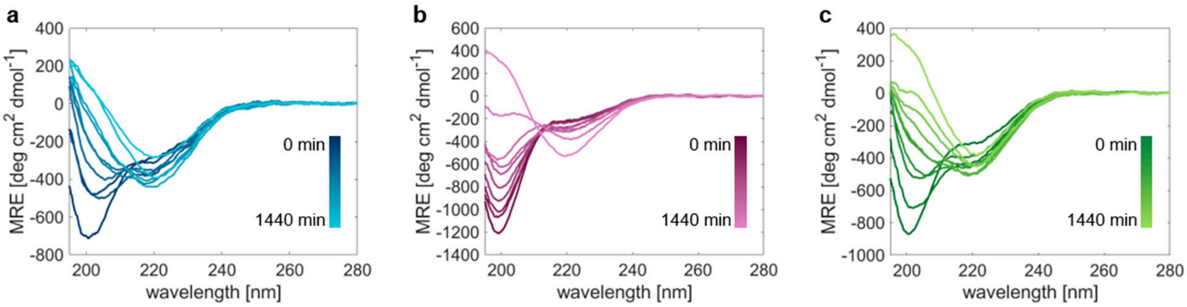

Figure S4. Time-resolved CD spectra of the spin-labeled aS variants. Spectra of (**a**) aS A27C-MTSSL, (**b**) aS A30P-A27C-MTSSL, and (**c**) aS A53T-A27C-MTSSL were acquired in real-time during the process of aggregation. The transition from random coil to β-sheet structure is clearly visible for all aS variants.

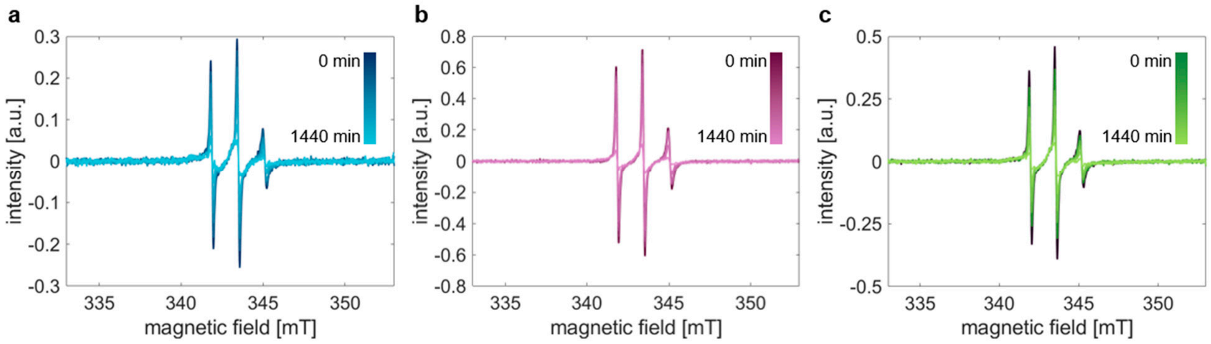

Figure S5. Time-resolved CW EPR spectra of the spin-labeled aS variants. Room-temperature spectra of (**a**) aS A27C-MTSSL, (**b**) aS A30P-A27C-MTSSL, and (**c**) aS A53T-A27C-MTSSL acquired in real-time during the process of aggregation. The rotational motion of the spin label is reduced upon progression of aggregation resulting in spectral broadening

References

1. Stoll, S.; Schweiger, A. EasySpin, a Comprehensive Software Package for Spectral Simulation and Analysis in EPR. *J. Magn. Reson.* **2006**, *178*, 42–55, doi:10.1016/j.jmr.2005.08.013.
